# Impacts of Stress and Risk Perception on Mental Health of College Students During the COVID-19 Pandemic: The Mediating Role of Coping Efficacy

**DOI:** 10.3389/fpsyt.2021.767189

**Published:** 2022-02-04

**Authors:** Fuming Xu, Long Huang

**Affiliations:** ^1^School of Education Science, Nanning Normal University, Nanning, China; ^2^School of Psychology, Jiangxi Normal University, Nanchang, China; ^3^School of Humanities and Management, Wannan Medical College, Wuhu, China

**Keywords:** coping efficacy, epidemic stress, risk perception, mental health, college students

## Abstract

**Object:**

In this study, we aimed to explore the influences of pandemic stress, risk perception, and coping efficacy on the mental health of Chinese college students during the COVID-19 pandemic.

**Methods:**

A nationally representative sample of Chinese college students (*N* = 3,381, *M*_*age*_ = 20.85, *SD*_*age*_ = 1.31) took part in an online survey during the COVID-19 pandemic. Correlation coefficients, structural equation modeling, and other statistical analysis methods were used for data analysis.

**Results:**

(1) The Chinese college students' pandemic stress and perceived pandemic risk were found to be moderate (3.51 ± 0.83, 3.45 ± 0.94), whereas their perceived infection risk was lower (2.10 ± 0.67). Their mental health during the COVID-19 pandemic was found to be good (3.80 ± 0.73). (2) The quality of their mental health was significantly and negatively associated with pandemic stress, perceived pandemic risk, and perceived infection risk. The level of their mental health was significantly and positively associated with coping efficacy, and their coping efficacy was significantly and negatively associated with pandemic stress, perceived pandemic risk, and perceived infection risk.

**Conclusion:**

Coping efficacy played a partial mediating role in the relationship between pandemic stress and mental health, coping efficacy played a partial mediating role in the relationship between perceived infection risk and mental health, and coping efficacy played a complete mediating role in the relationship between perceived pandemic risk and mental health. Our findings show the importance of fostering college students' coping efficacy to improve their mental health during the COVID-19 pandemic.

## Introduction

The COVID-19 pandemic broke out in late 2019 in Wuhan, China. The pandemic was officially recognized as one of the greatest “public health emergencies in the world” by the WHO on January 31, 2020, and it reached pandemic status throughout the world on March 11, 2020. As a major public health emergency, the COVID-19 pandemic has caused serious threats and heavy losses to health and lives all over the world. As of November 20, 2021, more than 257 million people had been infected worldwide with a death toll exceeding 5.15 million according to the WHO. In addition, the COVID-19 pandemic has caused panic, anxiety, and depression among those affected by it. This chain reaction triggered by negative emotion can be expected to further endanger the public's mental health, especially among children and adolescents (1–3). It is worth mentioning that a latest and global systematic review, which was conducted during the COVID-19 pandemic between January 1, 2020, and January 29, 2021, and included 204 countries and territories, showed that daily COVID-19 infection rates and reductions in human mobility were associated with an increased prevalence of major depressive disorder and anxiety disorders. Female subjects were affected more by the pandemic than male ones in terms of major depressive disorder and anxiety disorders, and younger subjects were more affected than older ones in terms of major depressive disorder and anxiety disorders (4). Therefore, it is particularly important to investigate mental health status and its influencing factors on young college students during the COVID-19 pandemic.

### Pandemic Stress, Risk Perception, and Mental Health

Besides the current COVID-19 pandemic, the SARS epidemic broke out in 2003, the H1N1 pandemic in 2009, and the Ebola epidemic in 2014, all of which caused serious losses of life and damage to health throughout the world. Therefore, researchers in academia conducted a series of empirical research on the relationship between stress response, risk perception, and mental health in the abovementioned major public health events. This research consistently found the impacts of stress and risk perception on mental health during these pandemic (5–10).

Specifically, a previous study on SARS explored the patterns and characteristics of Chinese college students' stress response and their levels of anxiety (SAS) and depression (SDS). The results show that panic was the most important element in the acute stress response related to SARS, followed by a defensive response and cognitive appraisal of the situation surrounding the epidemic, which played a moderating role. The stress response of college students had a significant impact on anxiety and depression (11). However, this study also found that the SAS and SDS could not be used to distinguish between the emotional responses of college students in high-incidence areas and non-high-incidence ones. It can be seen that, on the one hand, using a single self-rating scale of anxiety and depression might be problematic for accurately measuring the level of mental health (11). On the other hand, when facing major public health events, the public's mental health is directly related to their stress and risk perception, but most of the above studies mainly measured anxiety or depression. Therefore, future research should use more comprehensive measures of mental health and explore the causes and determinants of individual mental health during the pandemic (12–15).

### The Role of Coping Efficacy in the Relationship Between Stress, Risk Perception, and Mental Health

It is very important to explore the mediation between stress, risk perception, and mental health. Previous research shows that general self-efficacy plays an important role in the relationship between stress coping and mental health (16–18). Coping efficacy refers to an individual's belief in whether they can deal with the emotional environment and emotions aroused by a situation (19). Relative to general self-efficacy, coping efficacy is domain-specific (20). Tong (21) developed a coping efficacy questionnaire and compared the predictive power of both coping efficacy and general self-efficacy on college students' mental health during the SARS epidemic. The results show that coping efficacy plays a more important role than general self-efficacy in determining the severity of somatic symptoms, depression, and anxiety (21). In addition, other related studies find that coping efficacy was significantly and positively associated with individual stress coping and social adaption (22–24).

**Figure 1:**
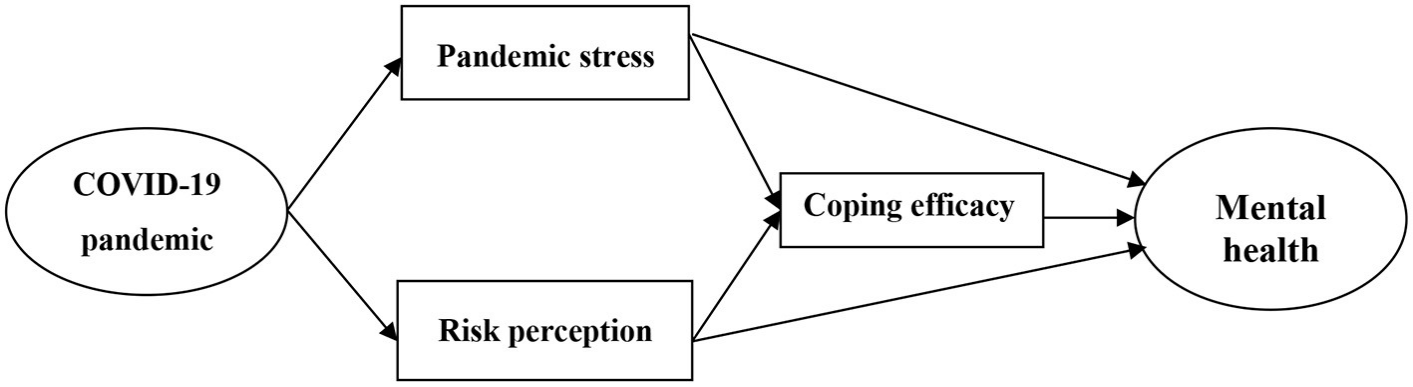
The relationship model of pandemic stress, risk perception, coping efficacy, and mental health.

Two conceptual frameworks guide such mediation hypotheses. According to the stress coping theory, the mandatory lockdown to control COVID-19 may be seen as a stressor, which may endanger college students' mental health (25, 26). Besides this, and according to self-efficacy theory, coping efficacy plays an important mediating role in the relationship between college students' stress, risk perception, and mental health during COVID-19 pandemic (18, 22). Therefore, this study attempts to construct a model of the relationship between pandemic stress, risk perception, coping efficacy, and mental health (Figure 1). As shown in Figure 1, college students may have experienced some degree of stress response and perceived pandemic risk when facing the outbreak of COVID-19 in China. On the one hand, college students' pandemic stress and levels of risk perception may have a direct impact on their mental health. On the other hand, college students' pandemic stress and levels of risk perception may also have an indirect impact on their mental health through the mediating role of coping efficacy. This is because coping efficacy can not only buffer the negative impacts of pandemic stress and risk perception on mental health (21, 23, 24), but also directly promote good mental health (19, 20). The objective of this study was to explore the relationship between college students' stress, risk perception and mental health during the COVID-19 pandemic. Based on this objective and hypothesis, we select a nationally representative sample of Chinese college students as participants and use a structural equation model (path analysis) to test the relationship between college students' pandemic stress, risk perception, coping efficacy, and mental health.

## Methods

### Participants and Design

An anonymous cross-sectional survey was conducted from February 11–March 1, 2020 (18–27 days after Chinese New Year and during winter vacation for college students) by using online questionnaires. A snowball sampling strategy was adopted with a focus placed on recruiting college students living in mainland China during the COVID-19 pandemic. The college students surveyed were from 28 provinces, including Guangdong, Guangxi, Anhui, Hubei, Zhejiang, Hunan, Beijing, Jiangxi, Shandong, Henan, and Heilongjiang. The average age of the college students was 20.85 ± 1.31 years.

The inclusion criterion was that the subjects needed to be full-time college students. The exclusion criteria included the following: (a) self-reported COVID-19 diagnosis (*n* = 13) and (b) failure to pass the internal consistency checks (*n* = 97). It was specified on the questionnaire that the return of the completed questionnaire implied that informed consent had been given. Ethical approval was obtained from the ethics committee of the corresponding author's affiliated university. The analyzed sample included 3,381 college students.

### Measures

#### General Health Questionnaire

The General Health Questionnaire (GHQ-12) is composed of 12 items and is considered to be the best mental health measurement tool, which is considered to have good reliability and validity (14, 27, 28). Likert 5-point scoring was used. The higher the score, the better the mental health. The data of 1,690 participants were used to conduct a confirmatory factor analysis on the GHQ-12. The chi-square value = 67.35, DF = 39, *P* = 0.09, chi-square value/DF = 1.73, GFI = 0.99, AGFI = 0.98, NFI = 0.98, IFI = 0.99, CFI = 0.99, RMSEA = 0.02. The Cronbach's α coefficient of the GHQ-12 was 0.88.

#### Pandemic Stress Assessment Questionnaire for COVID-19

Referring to the SARS stress study (29), four items were used to measure the pandemic stress felt by the population during the COVID-19 outbreak, which included questions such as “How much stress have you felt during the COVID-19 pandemic?” Likert 5-point scoring was used. The higher the score, the more the perception of pandemic stress. First, the data of 1,691 subjects were used to analyze the exploratory factors of pandemic stress in relation to the four items, and one factor with a characteristic root >1 was extracted, whereas the interpretation rate was 69.13%. Then, the data of the other 1,690 subjects were used to analyze the confirmatory factors of pandemic stress in relation to the four items, and it was found that the chi-square value = 2.57, DF value = 1, *P* = 0.16, chi-square value/DF = 2.19, GFI = 1.00, AGFI = 0.98, NFI = 1.00, IFI = 1.00, CFI = 1.00, and RMSEA = 0.04. The Cronbach's α coefficient was 0.85.

#### Risk Perception Self-Rating Questionnaire for COVID-19

Referring to the Xie et al. (6) risk perception self-rating questionnaire (SARS) combined with knowledge of the COVID-19 pandemic situation, a seven-item risk perception self-assessment questionnaire, was developed for this study. Likert 5-point scoring was used. First, an exploratory factor analysis of risk perception was conducted with the data of 1,691 subjects, and two factors with feature roots >1 were extracted with a cumulative interpretation rate of 67.94%. Factor 1 can be called “perceived pandemic risk”; its explanation rate is 43.86%. Factor 2 can be called “perceived infection risk”; its explanation rate is 24.09%. Then, the data of the remaining 1,690 subjects were used to conduct a confirmatory factor analysis on the risk perception self-assessment questionnaire. The chi-square value = 20.36, DF = 8, *P* = 0.07, chi-square value/DF = 2.55, GFI = 0.98, AGFI = 0.97, NFI = 0.98, IFI = 0.98, CFI = 0.99, RMSEA = 0.04. The Cronbach's α coefficient of the pandemic risk subscale was 0.81, the Cronbach's α coefficient of the infection risk subscale was 0.72, and the Cronbach's α coefficient of the entire questionnaire was 0.80.

#### Coping Efficacy Questionnaire

The questionnaire was developed by Tong (21) and had a total of 17 items, which were divided into three dimensions: competence, confidence, and cognitive appraisal. The questionnaire is often used to measure the evaluation of an individual's coping ability in a state of stress. We used Likert-style four-point scoring. The higher the score, the higher the coping efficacy. The confirmatory factor analysis of the coping effectiveness questionnaire showed that the chi-square value = 76.83, DF = 46, *P* = 0.06, chi-square value/DF = 1.67, GFI = 0.98, AGFI = 0.97, NFI = 0.98, IFI = 0.99, CFI = 0.99, and RMSEA = 0.03. The Cronbach's α coefficient of competence was 0.93, the Cronbach's α coefficient of confidence was 0.84, the Cronbach's α coefficient of cognitive appraisal was 0.72, and the Cronbach's α coefficient of entire questionnaire was 0.86.

### Statistical Analysis

Independent *t*-tests were used to test the significance of between-group differences. Pearson correlations were used to test the associations between mental health and its related influencing factors. A structural equation model (path analysis) with full information likelihood estimation was used to test the hypothesized mediation model for mental health. Tests for the direct, indirect, and total effects were based on 2,000 bootstrapped samples. Effect estimates and bias-corrected 95% confidence intervals (CI) were derived. The indices of good fit included the root mean square error of approximation (RMSEA) < 0.06, comparative fit index (CFI) > 0.95, etc. Analyses were conducted using SPSS 22.0 and AMOS 22.0. A two-sided *p* below 0.05 was considered statistically significant.

## Results

### Sample Characteristics

First, college students' sociodemographic characteristics, pandemic stress, risk perception, and mental health were documented in Table 1. The level of pandemic stress and perceived pandemic risk were found to be moderate, whereas the perceived infection risk appeared to be lower. The level of mental health of college students during the COVID-19 pandemic was found to be good.

**Table 1:** Descriptive statistics of the entire sample (*n* = 3,381).

**Variables**	***M* ±SD or *n* (%)**
**Background variables**	
Age (years)	20.85 ± 1.31
**Sex**	
Male	1,364 (40.34)
Female	2,017 (59.66)
**Grade**	
First-year	861 (25.47)
Second-year	852 (25.20)
Third-year	840 (24.85)
Fourth-year	828 (24.48)
Pandemic stress	3.51 ± 0.83
**Risk perception**	
Pandemic risk	3.45 ± 0.94
Infection risk	2.10 ± 0.67
Mental health	3.80 ± 0.73

Second, we tested for gender differences in relation to the college students' pandemic stress, risk perception, and mental health. The results showed that there were significant gender differences in the data (*t* = −11.98, *p* < 0.001). Female college students (3.22 ± 0.82) felt higher levels of pandemic stress than male college students (2.85 ± 0.87). There were significant gender differences in terms of the perceived pandemic risk (*t* = −7.28, *p* < 0.001). The perceived pandemic risk of female college students (3.53 ± 0.90) was higher than that of male college students (3.28 ± 0.99). There were significant gender differences in perceived infection risk levels (*t* = −4.35, *p* < 0.001). The perceived infection risk of female college students (2.14 ± 0.66) was higher than that of male college students (2.03 ± 0.69). There was no gender-based difference in college students' mental health (*t* = 1.22, *p* > 0.05).

### Relationship Between Stress, Risk Perception, Coping Efficacy, Mental Health

First, we tested the correlations between college students' pandemic stress, risk perception, coping efficacy, and mental health. The results are shown in Table 2. It can be seen that there are significantly negative correlations between college students' mental health and pandemic stress, pandemic risk, and infection risk, respectively, and that there is a significantly positive correlation with coping efficacy.

**Table 2:** Correlations between pandemic stress, risk perception, coping efficacy, and mental health.

	**1**	**2**	**3**	**4**	**5**
1. Pandemic stress	1				
2. Pandemic risk	0.49^***^	1			
3. Infection risk	0.25^***^	0.18^***^			
4. Coping efficacy	−0.17^***^	−0.05^**^	−0.38^***^	1	
5. Mental health	−0.33^***^	−0.15^***^	−0.30^***^	0.59^***^	1

** *p < 0.01,*

*** *p < 0.001*.

**Figure 2:**
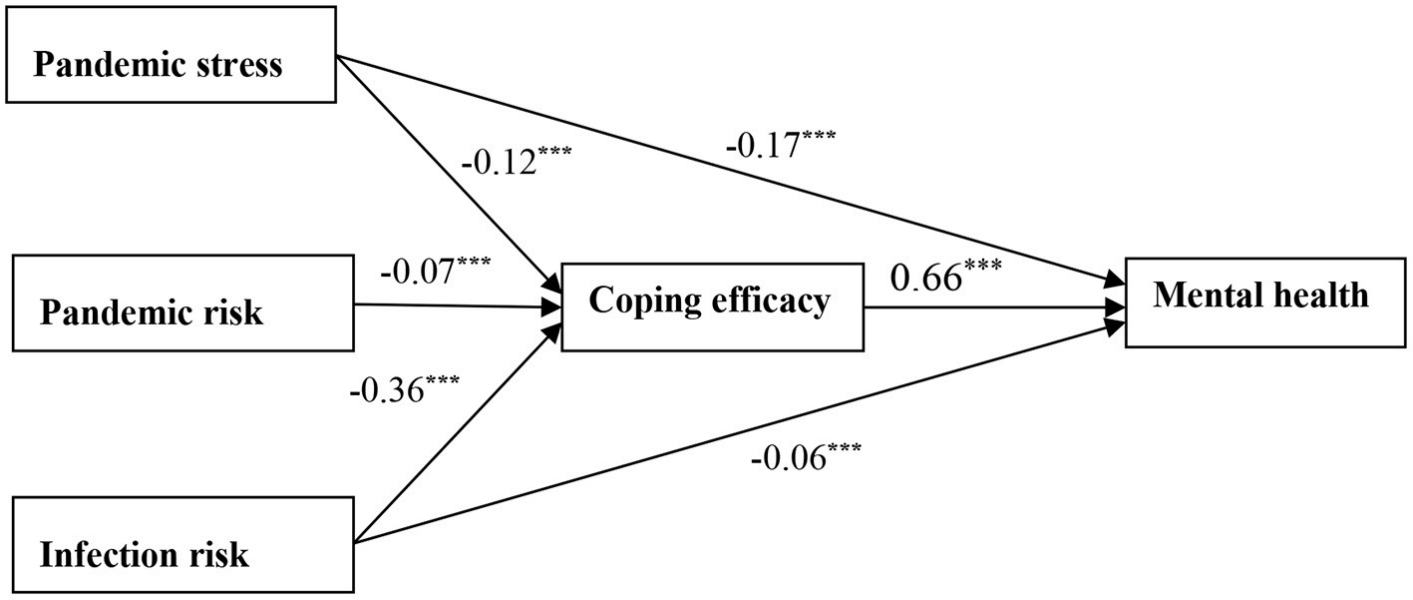
Relationship between pandemic stress, risk perception, coping efficacy, and mental health. ***p < 0.001.

Second, we constructed a model of the relationship between college students' pandemic stress, risk perception, coping efficacy, and mental health by using a structural equation model (path analysis) (Figure 2). The chi-square value = 0.93, DF = 1, *P* = 0.33, chi-square value/DF = 0.93, GFI = 1.00, AGFI = 1.00, NFI = 1.00, IFI = 1.00, CFI = 1.00, and RMSEA = 0.01. On the one hand, college students' pandemic stress and perceived infection risk had a directly negative predictive effect on their mental health. On the other hand, coping efficacy played a partial mediating role in the relationship between pandemic stress and mental health; coping efficacy played a partial mediating role in the relationship between perceived infection risk and mental health; and coping efficacy played a complete mediating role in the relationship between perceived pandemic risk and mental health. In addition, the total effect of each variable on mental health was 54% of which the total direct effect was 23%. The effect of each variable on coping efficacy was 16%.

## Discussion

### College Students' Pandemic Stress, Risk Perception, and Mental Health

Overall, Chinese college students' levels of pandemic stress and risk perception during the COVID-19 pandemic were relatively low, and their mental health was found to be good. Further, the gender-difference test showed that the levels of pandemic stress and risk perception among female college students were slightly higher than those of male college students. The results were partially consistent with those of Ma et al. (3) and Santomauro et al. (4), which showed that female subjects were affected more by the pandemic than male ones in terms of depression and anxiety. This might be the case because female college students are usually more sensitive to stressful situations. In addition, owing to the need for pandemic control, college students were “forbidden” to leave their homes and could not get together with classmates, relatives, or friends, which might have a greater impact on female college subjects (13, 30). However, although the subjects generally felt some degree of pandemic stress and perceived some risks, their mental health appeared to be good. The results were consistent with those of the related study by Xin et al. (8). In addition, the result was partially consistent with another study, which showed mental health and loneliness reported by young people were lower in China than that in the United Kingdom during the COVID-19 pandemic (14). On the one hand, this might be the case because the Chinese Spring Festival and winter vacation played a double-buffering role during the outbreak of COVID-19 in China. On the other hand, these results might stem from the fact that Chinese people responded positively and cooperated with the authorities in efforts to control the COVID-19 pandemic, so the outbreak was effectively controlled within a short period (31). This context might have played an important role in maintaining college students' mental health and alleviating the negative impacts of stress and infection risk on their mental health (8, 14).

### The Role of Coping Efficacy in Relationship Between Stress, Risk, and Mental Health

First, we found that college students' pandemic stress and perceived infection risk had a significantly and negatively predictive effect on their mental health. Higher levels of pandemic stress and perceived infection risk among the subjects were not conducive to maintaining good mental health. These results were consistent with those of the relevant studies conducted by Xie et al. (6) and Tong (11) in relation to the SARS epidemic. The results were partially consistent with those of Wen et al. (7) and Zhang et al. (10), which showed that people's perceived risk and the perceived stress of the COVID-19 pandemic had a negative predictive effect on their anxiety levels. These results prove that both pandemic stress and perceived risk were two important factors affecting college students' mental health. Therefore, it is important to provide psychological counseling and promote support from families, schools, and society for affected college students during the COVID-19 pandemic to help them maintain good mental health (13, 28, 30).

Second, we found that college students' pandemic stress and perceived infection risk had an indirect predictive effect on mental health through the partial mediating role of coping efficacy. This result was consistent with those of Wang et al. (23) and Ma et al. (24), which showed that college students' coping efficacy had a greatly positive impact on their mental health. Therefore, our finding demonstrates the importance of fostering coping efficacy to enhance college students' mental health. In addition, we found that coping efficacy played a complete mediating role in the relationship between perceived pandemic risk and mental health, which might indicate that there was no direct relationship between pandemic risk and mental health. College student's perceived pandemic risk indirectly affected mental health through coping efficacy. It can be seen that, although both concepts belong to the domain of risk perception, perceived pandemic risk and perceived infection risk could be distinct psychological constructs that have different effects on mental health, which is worthy of further exploration in future research.

## Limitations and Future Research

Our survey belongs to the domain of quantitative research and lacks qualitative analysis. In the future, in-depth interviews could be combined with case studies and follow-up research. In addition, our results draw on cross-sectional data using structural equation model. Although we recruited a large sample, this design cannot be used to draw conclusions about causal relationship. Future research will require the use of a longitudinal survey or intervention design. Finally, the Chinese context of our study and the present global situation differed in many ways in terms of aspects, such as social distancing restrictions. It is important to validate our results by comparing them with results obtained in other contexts and identify similarities and differences with other countries and regions.

## Practical Implications

First, the current research further reveals the impact of the COVID-19 pandemic on the mental health of young college students and the causes and determinants of mental health problems. This will help to carry out targeted interventions for the mental health of college students as well as interventions to treat those who develop a mental disorder (4). Second, in view of the cultivable characteristics of self-efficacy, those responsible for the design and organization of college education and extracurricular activities should consider providing more opportunities for college students to engage in exercise with the aim of continuously improving students' coping efficacy, which will not only help improve their mental health, but also greatly enhance their learning and lives (18, 23, 24).

## Conclusion

The results show that coping efficacy was one potential mechanism mediating the relationship between pandemic stress, risk perception, and mental health. Coping efficacy played a partial mediating role in the relationship between pandemic stress, perceived infection risk, and mental health; Additionally, coping efficacy played a complete mediating role in the relationship between perceived pandemic risk and mental health.

## Data Availability Statement

The raw data supporting the conclusions of this article will be made available by the authors, without undue reservation.

## Ethics Statement

The studies involving human participants were reviewed and approved by Ethics Committee of School of Educational Sciences, Nanning Normal University. The patients/participants provided their written informed consent to participate in this study.

## Author Contributions

LH and FX designed this study. FX wrote the paper. All authors contributed to the article and approved the submitted version.

## Funding

This study was supported by the National Natural Science Foundation of China (Grant Nos. 72164028 and 71971103) and MOE (Ministry of Education in China) Project of Humanities and Social Sciences (Grant No. 20YJC190006).

## Conflict of Interest

The authors declare that the research was conducted in the absence of any commercial or financial relationships that could be construed as a potential conflict of interest.

## Publisher's Note

All claims expressed in this article are solely those of the authors and do not necessarily represent those of their affiliated organizations, or those of the publisher, the editors and the reviewers. Any product that may be evaluated in this article, or claim that may be made by its manufacturer, is not guaranteed or endorsed by the publisher.
